# Older adults in the digital health era: insights on the digital health related knowledge, habits and attitudes of the 65 year and older population

**DOI:** 10.1186/s12877-023-04437-5

**Published:** 2023-11-27

**Authors:** Zsuzsa Győrffy, Julianna Boros, Bence Döbrössy, Edmond Girasek

**Affiliations:** Institute of Behavioural Sciences, Faculty of Medicine, Semmelweis University, Nagyvárad tér 4. 20th floor, Budapest, H-1089 Hungary

**Keywords:** Older adults, Digitalization, Online information seeking, Digital solutions, Bio-psycho-social-digital model

## Abstract

**Background:**

The COVID-19 pandemic has increased internet use by older age groups to an unprecedented level in Hungary mirroring the general tendency in the total population. Nevertheless, international trends indicate that this group is less likely to use digital health technologies than younger ones. The aging population raises the question of successfully integrating elderly people into the digital health ecosystem. Our research aim is to investigate the digital health usage patterns and attitudes of the population aged 65 and over through a representative sample.

**Methods:**

A national representative questionnaire survey was conducted by telephone (CATI), interviewing 1723 respondents. Within this sample we examined 428 people in the over-65 age group, 246 in the 65–74 age group and 182 in the over-75 age group. Predictors of demand for digital solutions were tested using binary logistic regression model.

**Results:**

50.8% of people aged 65–74 and 37.1. % of people aged 75 + use the internet for health-related purposes, mostly to access websites. 85% of respondents in 65–74 and 74% in 75 + age group have used more than one digital health device and around 70% of both age groups have a need for more than one digital solution. 90.2% (64–75 age group) and 85.7% (75 + age group) of respondents are familiar with e-prescription, 86.4% and 81.4% of them use it. 77.1% of 65–74-year-olds have heard of and nearly half 45.5% have used online appointment. More than half (52.7%) of the respondents in this age group have heard of and used electronic transmission of medical records and data. A similar proportion has heard about and used apps: 54.3% has heard of them, but only 17.3% has used them. The multivariate analyses emphasized that the need for digital solutions increases with the level of education and the more benefits one perceives in using digital solutions.

**Conclusion:**

Our research has shown that the senior age group has measurable needs in the field of digital health, so helping them on this journey is in the interest of the whole health ecosystem. Their high level of interest is indicated by the fact that more than a fifth of older adults would like to have access to between 7 and 10 of the maximum number of digital devices available. The differences between the two age groups - with younger people being more open to digital solutions and using them more - and the fact that the under 65s are better adapted digitally in all respects, raises the possibility that the specific trends in digital health for older people may virtually disappear in 10 years’ time (when the under 65s now enter this age group).

**Supplementary Information:**

The online version contains supplementary material available at 10.1186/s12877-023-04437-5.

## Background

Digitalisation in health was emerging as an important new trend before COVID-19, but has been boosted significantly by the pandemic [1]. At the same time, the issue of digital health inequalities has become increasingly important. This is the so-called digital health paradox, i.e. the unequal way in which social groups can take advantage of the digital transformation and opportunities. [2]. These issues are of particular relevance for the ageing population. This is a demographic group with a relatively high proportion of people living with chronic illness, disability and isolation. It could be a major target for digital health solutions. However, the adaptation of digital health solutions is lower in this age group globally compared to younger people [3–5].

It is worth emphasising that there are several difficulties in defining an ageing population, as we may distinguish between biological/chronological/physical/social/cultural ages [6]. In the present analysis, we have used a chronological approach (dividing 65–74-year-olds and those aged 75 and over), with the limitation that, as with international trends, older people are not a homogeneous group: we can observe large differences in demographic characteristics (age or educational levels) or in participation in the labour market or income status [7]. This age group is also quite heterogeneous in terms of their attitude towards technology: in addition to prior experience and physical/mental health, adaptation is also significantly influenced by social relationships [8].

The COVID-19 pandemic has increased internet use among older age groups to an unprecedented level in Hungary [9, 10] mirroring the general tendency in the total population. Nevertheless, international trends indicate that this group is less likely to use digital health technologies than the younger ones. This is especially so among less educated older people and those living in smaller settlements [11]. In terms of access, skills and engagement, research reports lower trends in this age group, but higher rates of “technostress” and of perceiving these technologies as “useless” [12–15]. Technostress is defined as the stress that people experience as a result of their use of information and communication systems and technologies [16].

According to the systematic literature review of Shi, Ma, Zhang and Chen on the internet use of elderly people in China, gender, educational attainment, socioeconomic status, physical and psychological conditions are all influencing factors in internet use [17]. Other research evidence seems to suggest that the digital gender divide is stronger among the elderly population than among the younger age groups. While in the UNECE region internet use gender parity has been achieved in the 24–54 year age group, less women in the 55–75 year group use the internet than men [18]. When it comes to digital skills, the disadvantage of women in the elderly category is even more pronounced. In EU countries, only half as many women between the ages of 55 and 74 have basic or just above basic digital skills than men in this age group or younger women [19]. Results are not conclusive, nevertheless. The study of Cao et al. in China [20] showed that older people with high levels of social connection, social participation, trust, reciprocity, and cohesion are more likely to prefer eHealth literacy in later life, but found no gender difference.

Various digital technologies can be of significance for this segment. Examples may include those used in monitoring and tracking health conditions and diseases, accessing relevant health information, and various telemedicine solutions which can provide a safe and convenient way to connect with caregivers. This could facilitate health promotion and illness prevention, greater autonomy and independence for ageing people [21, 22]. The internet also provides a means of communication and greater degree of social connectivity for this age group, reducing isolation and increasing well-being, meaning greater autonomy and social connectedness [23, 24].

Defining the concept of “digital health” can be challenging. In 2020, Fatehi and colleagues conducted a comprehensive review of nearly 1500 articles, revealing approximately 95 distinct scientific and lay definitions of the concept [25]. Some of these focused on the technological aspects of digital health, (digital health for example encompasses elements like mHealth, telehealth, telemedicine, and wearable devices.) In contrast, others place greater emphasis on the human dimension, highlighting aspects such as participatory medicine, the evolution of the doctor-patient relationship, and the democratization of healthcare.In the context of the present study, we delve into the utilization of digital health and the cultural shifts it brings about. Within this conceptual framework, digital health is not solely a revolution in terms of technology; it also represents a cultural and social transformation. This transformation extends to the reconfiguration of the doctor-patient relationship, changes in decision-making processes, and the management of health [26].

Hungary ranks 22^nd^ among the 27 EU Member States in the Digital Economy and Society Index (DESI) [27]. The country performs well on broadband connectivity. It remained a leader in the take-up of at least 1Gbps broadband, as 22% of households subscribed to such a service in 2021 compared with 7.6% in the EU. As for human capital on the other hand, only 49% of individuals have at least basic digital skills, which is below the EU average of 54%. 3.1% of graduates studied ICT (EU average: 3.9%) [27]. According to the data of the Hungarian Central Statistical Office, in 2020 88% of households had internet access, which is slightly below the EU average [28]. A study done by the National Media and Infocommunications Authority found that 34% of people aged over 65 (1.9 million people) use the Internet in Hungary. Of people living alone in this age group 31% are users while 42% of those living with a partner use the internet in this population [29]. Those who are Internet users are active social media users, too. 2 out of 3 use Facebook, and every second Internet user uses some chat programme (written or oral) to keep in touch with acquaintances. This is a 10% point increase in a year [30].

21% of the population of Hungary qualified as older adults on 1^st^ October 2022, and between 2011 and 2022, the number of people aged 65 and over increased by 318,000, or 19% [31]. The growth of the elderly age group raises the question of how to successfully integrate them into the digital health ecosystem. Our research aims to investigate the digital health usage patterns and attitudes of the 65 and over population through a representative sample. Identifying the social factors that may contribute to the increase in demand for different digital health solutions is an important research topic. By digital health solutions we mean health-related information on the Internet, the use of different digital communication channels (email, social media channels), telemedicine solutions (e-prescription, online appointment booking, telehealth) and the use of specific technologies (sensors, smart devices, apps).

## Methods

Within the framework of the research programme “E-doctors and e-patients in Hungary: the role and opportunities of digitalisation in health care” - OTKA-FK 134,372, funded by the National Research Centre for Health Research and Innovation (NKFIH), a national representative questionnaire survey was conducted in Hungary by telephone (CATI), interviewing 1723 respondents. The sample was selected using stratified sampling in terms of gender, age, type of municipality and educational level. It is representative of the adult population of Hungary. Data collection was carried out by Ipsos Zrt. between 5 and 13 October 2021. The sampling frame was 12,000 persons, randomly selected from an open telephone enquiries database, and 8000 persons were selected as a reserve sample. 11,733 respondents refused to complete the survey and 1293 dropped out, but most of this was due to the sampling quota. The outreach was 80% mobile and 20% land-line telephone. Correction weighting was applied to the data to improve representativeness.

The research has an ethical licence. The number is IV-10927-1/EKU.

### Measuring instruments

The main blocks of the 25-question, 15-minute, self-developed questionnaire were: sociodemographic data, presence of chronic disease, frequency of internet use for health purposes and type of searches, knowledge and use of digital health technologies, needs for digital technologies, and positive and negative attitudes towards the use of digit.al health solutions. Our questionnaire is available in the Supplementary file 1.

Among the sociodemographic variables, we examined the two-category variable gender, age 65–74 and 75 and over, type of settlement (capital, county-seat, city, village/town), marital status (living with partner yes/no) and the variable presence of chronic disease (having a chronic disease yes/no).

The survey questionnaire contained the following question about health-related Internet use habits : “How often do you use the internet to collect health related information?”. The possible five response categories (daily, weekly, monthly, rarer, never) for analytical purposes were grouped into two categories: (1) at least monthly (2) rarer or never.

As for Internet platforms used for health-related information or news, the questionnaire had the following options: websites, blogs, podcasts, social media sites, online communities, video content sharing sites, scientific, literature search engines, medical professional sites.

Regarding perceptions of medical attitudes to online information, the following question was asked: “How do you perceive doctors’ attitudes towards patients who use the internet for information?” For the answers a five points Likert scale was used (1 – totally negative 5 – totally positive).

We also asked if respondents use the Internet when they see a doctor with a health problem (answer options: yes before going to the doctor, yes after going to the doctor, yes before and after going to the doctor, no).

We asked the following question about the use of different digital technologies: “Which of the following have you heard of, used or would like to use?” (answer options: online appointment booking, e-prescription, data/information transmission, social media for health, applications, telemedicine, smart devices for health monitoring).

The question “Which of the following options do you use and which would you use if you had the opportunity?“ was used to assess the needs for digital health solutions (response options: use, do not use, but would use, if you had the opportunity, would not use). At the time of analysis, the needs for digital health were further grouped into 3 categories: doctor-patient communication and information (email, use of social media channels, website recommendation), digital administration (transmitting records and pictures, online appointment request, forwarding electronic documentation to the doctor), telemedicine solutions (remote consultation, sensor, app, smartphone tracking by the doctor).

The advantages/disadvantages of different digital solutions were selected by the respondents from a list in the questionnaire (see supplementary file 2). In the analysis, a cumulative variable (with values ranging from 0 to 11) was created from the advantages/disadvantages question.

What do you think may be the potential *positive consequences* for society from using digital health solutions (e.g. apps on smartphones, smart watches, smart bands, other sensors?

**Table:** 

1.	Improves the convenience of health care (e.g. you can get care faster)
2.	Improves the safety of care
3.	Helps involve patients in the process of care
4.	It is comfortable
5.	Limits the number of in person doctor-patient
6.	Saves time
7.	Patients can get health care quicker
8.	Patients are more involved in the process of health care
9.	You can get better quality care
10.	Limits the possibility of malpractice
11.	Improves doctor-patient communication

## Potential negative effects of using digital health solutions

What do you think may be the potential *negative consequences* for society of using digital health solutions (e.g. apps on smartphones, smart watches, smart bands, other sensors?)

**Table:** 

1.	Health care becomes worse quality
2.	It frustrates doctors and patients (e.g. because of technical difficulties)
3.	Decreases patient satisfaction
4.	May lead to overdiagnosis (minor disease is screened which may lead to an increase of cases being treated and hence to an increased workload for the healthcare system)
5.	Patients misinterpret the health data they receive
6.	Faulty technology may endanger the healing of the patients
7.	Personal data are not safe
8.	The administrative burden of doctors increases
9.	The risk of medical burnout increases
10.	Health care becomes more impersonal
11.	Other……:

In the logistic regression, we collected information on openness to digital technologies and their intent to use them. A two category variable was created (0–1) as dependent variable from the variable of the question “How much digital technology would you need?“ (0–7 types of technology): 0 means no need for digital health technology, and 1 value means that respondent has a need to use one or more digital health technologies. The control variables were selected based on visible relations in the results of two-dimensional analyses (crosstabulations). These variables were associated with sociodemographic aspects such as gender, type of municipality, educational attainment, and the presence of chronic illness. Additionally, they were linked to attitudes towards digital technologies, specifically how much advantage or disadvantage of digital technologies were perceived by the respondents.

### Statistical methods

Data were analysed using IBM Statistics statistical data analysis software [32]. Statistical data processing included distributions, cross tabulations, chi-square tests, and comparing means with ANOVA. Significant (p < 0.05) correlations and differences are highlighted in bold in the tables and non-significant results are marked in all cases without bold highlighting. Predictors of demand for digital solutions were tested using binary logistic regression analysis. In the logistic regression, we simultaneously entered gender, education, type of settlement, presence of chronic disease and perceived benefits and disadvantages of different digital health solutions, while the dependent variable was the presence of need for digital technologies.

## Results

### Demography

There were 428 people in the-65 year and older age group, 246 in the 65–74 age group and 182 in the over-75 age group. Women were 59.3% of the 65–74 age group, compared to 72% of the 75 + age group. 55.5% of those aged 65–74 have at most primary education, compared to 58.8% of those aged 75+. University/college graduates represented 12.7% and 10.4% of these two age groups respectively. Both age groups had significantly higher rates of female and lower educational qualifications.

As for residency type, the highest proportions lived in cities (34.7% and 32.4%), followed by villages/towns (31.8% and 25.8% respectively) while the proportions of those living in the capital (16.7% and 25.8%) and county seats (16.7% vs. 15.9%) were similar.

While 54.3% of 65–74-year-olds lived with a partner, the ratio was 30.2% for those aged 75 and over. The two age groups had almost similar rates of chronic diseases (76.7% and 73.6%): around three quarters of the age group had chronic disease. Both the proportion of people living alone and the number of people with a chronic illness were significantly higher in older age groups (Table 1).

**Table 1:** Socio-demographical distribution of the respondents

		18–64 years old	65–74 years old	75 + years old
Gender				
**Male**	**n**	**651**	**100**	**51**
	**%**	**50.3%**	**40.7%**	**28.0%**
**Female**	**n**	**643**	**146**	**131**
	**%**	**49.7%**	**59.3%**	**72.0%**
**Total**	**n**	**1294**	**246**	**182**
Level of education				
**Elementary school or lower**	**n**	**231**	**136**	**107**
	**%**	**17.8%**	**55.5%**	**58.8%**
**Vocational school / skilled worker**	**n**	**348**	**27**	**12**
	**%**	**26.9%**	**11.0%**	**6.6%**
**High school**	**n**	**456**	**51**	**44**
	**%**	**35.2%**	**20.8%**	**24.2%**
**Higher education**	**n**	**260**	**31**	**19**
	**%**	**20.1%**	**12.7%**	**10.4%**
**Total**	**n**	**1295**	**245**	**182**
Type of residence				
Budapest	n	223	41	47
	%	17.2%	16.7%	25.8%
County capital, large city	n	240	41	29
	%	18.5%	16.7%	15.9%
Other city	n	459	85	59
	%	35.5%	34.7%	32.4%
Village	n	372	78	47
	%	28.7%	31.8%	25.8%
Total	n	1294	245	182
Lives alone / with a partner				
**Lives alone**	**n**	**460**	**112**	**127**
	**%**	**35.6%**	**45.7%**	**69.8%**
**Lives with partner**	**n**	**832**	**133**	**55**
	**%**	**64.4%**	**54.3%**	**30.2%**
**Total**	**n**	**1292**	**245**	**182**
Do you have chronic disease				
**No**	**n**	**797**	**57**	**48**
	**%**	**61.6%**	**23.3%**	**26.4%**
**Yes**	**n**	**497**	**188**	**134**
	**%**	**38.4%**	**76.7%**	**73.6%**
**Total**	**n**	**1294**	**245**	**182**

### Internet use and health information searching

The population survey shows that the likelihood of using the internet for health information decreases with age: while only one in six people under 65 say they do not use the internet for health information at all (16.5%), this is true for almost half (49.2%) of people aged 65–74 and almost two thirds (62.9%) of people aged 75 and over. Overall, 54.0% of the population aged 65–74 use the internet for any purpose, compared to 30.3% of those aged 75+.

When looking at the results for health-related information seeking, the proportion of people aged 65–74 who have a relative, friend or family member to help them find information is 21.3%. This percentage is 35.4% in the 75 + age group, while it is 3.0% in the 18–64 age group.

Nearly half (49.2%) of 65–74-year-olds never use the internet to find health and illness related information, compared to a higher rate of 62.8% for the older age group. 13.8% of 65–74-year-olds use 1–2 sources and 37% use more than 3 sources for information. Among the 75 + age group, the proportion of people who use 1–2 or more sources to find information on health-related issues is 14.2% and 23.0% respectively (Table 2).

**Table 2:** How many internet sources do you use to find health-related information? by age groups

Number of internet sources		18–64 years old	65–74 years old	75 + years old
**0**	**n**	**214**	**121**	**115**
	**%**	**16.5%**	**49.2%**	**62.8%**
**1–2**	**n**	**317**	**34**	**26**
	**%**	**24.5%**	**13.8%**	**14.2%**
**3 or more**	**n**	**763**	**91**	**42**
	**%**	**59.0%**	**37.0%**	**23.0%**
**Total**	**n**	**1294**	**246**	**183**
	**%**	**100.0%**	**100.0%**	**100.0%**

While women use more internet sources on average among those under 65, there is no significant difference between the two genders among those who are 65 and over. However, education is an important factor: those with lower education use significantly fewer internet sources for information, both in the 65–74 and 75 + age groups **(**Table 3**)**.

**Table 3:** How many internet sources do you use to find health-related information? by age and educational attainment

			Elementary school or lower	Vocational school / skilled worker	High school	Higher education
**18–64 years old**	**How many internet sources do you use to find health-related information?**	**0**	**77**	**72**	**48**	**17**
			**33.3%**	**20.7%**	**10.5%**	**6.5%**
		**1–2**	**60**	**93**	**89**	**75**
			**26.0%**	**26.7%**	**19.6%**	**28.8%**
		**3 or more**	**94**	**183**	**318**	**168**
			**40.7%**	**52.6%**	**69.9%**	**64.6%**
	**Total**		**231**	**348**	**455**	**260**
			**100.0%**	**100.0%**	**100.0%**	**100.0%**
**65–74 years old**	**How many internet sources do you use to find health-related information?**	**0**	**88**	**9**	**17**	**7**
			**64.7%**	**33.3%**	**33.3%**	**22.6%**
		**1–2**	**17**	**5**	**6**	**6**
			**12.5%**	**18.5%**	**11.8%**	**19.4%**
		**3 or more**	**31**	**13**	**28**	**18**
			**22.8%**	**48.1%**	**54.9%**	**58.1%**
	**Total**		**136**	**27**	**51**	**31**
			**100.0%**	**100.0%**	**100.0%**	**100.0%**
**75 + years old**	**How many internet sources do you use to find health-related information?**	**0**	**80**	**7**	**23**	**5**
			**75.5%**	**58.3%**	**52.3%**	**26.3%**
		**1–2**	**14**	**4**	**3**	**4**
			**13.2%**	**33.3%**	**6.8%**	**21.1%**
		**3 or more**	**12**	**1**	**18**	**10**
			**11.3%**	**8.3%**	**40.9%**	**52.6%**
	**Total**		**106**	**12**	**44**	**19**
			**100.0%**	**100.0%**	**100.0%**	**100.0%**

#### Health-related information seeking habits and physicians perceived attitudes towards finding information on the internet

In both age groups, websites, social media channels and medical search sites are the most popular sources of information, but the 65–74 age group has a significantly higher proportion of people who get information from websites (58.9% vs. 37.6% p < 0. 001) from websites, and social media channels (50% vs. 30.4% p < 0.003), online patient community sites (32.9 vs. 14.7 p < 0.001), video content sharing sites (41.1 vs. 22.5 p < 0.003). There is no difference in the use of medical search sites between the two groups.

We also asked how they perceived their physicians’ attitudes towards finding information on the internet. For this question, more than half (52.5%) of those aged 65–74 perceived their doctors to be supportive (4 or 5 value on the scale of 1–5), compared with 43.3% of those aged 75+. There is no significant difference in terms of gender in any age group, but in terms of education, both in the 18–64 and 65–74 age groups, as education increases, there is a higher proportion of perceived refusal and a lower proportion of perceived support. For the oldest age group, no significant relationship is observed by education (Table 4).

**Table 4:** How do you perceive the attitude of doctors towards patients who find information on the Internet? by age groups

		18–64 years old	65–74 years old	75 + years old
**dismissive**	**n**	**255**	**13**	**9**
	**%**	**26.5%**	**10.7%**	**15.0%**
**neutral**	**n**	**370**	**45**	**25**
	**%**	**38.4%**	**36.9%**	**41.7%**
**supportive**	**n**	**338**	**64**	**26**
	**%**	**35.1%**	**52.5%**	**43.3%**
**Total**	**n**	**963**	**122**	**60**
	**%**	**100.0%**	**100.0%**	**100.0%**

Respondents were also asked whether they used the internet to find information when they see a doctor. We found that for the most senior age group 78.0% do not use internet resources when consulting a physician in person. For those aged 65–74, the proportion is lower at 58.7%, and for age 18–64 this proportion is 37.8.

#### Knowledge and use of digital health tools

When asked what digital health solutions they have heard of (online appointment booking and referral request; prescription, e-prescription; transmission of data and findings; social media related to health information; applications (e.g.: sleep monitoring, blood sugar diary, symptom diary, etc.); telemedicine; smart devices, sensors (e.g.: smart watch, smart scale, pulse oximeter)); the highest proportions in both old age groups are those who have heard of 4–5 digital health solutions (37.7% in both groups), while the proportion who have not heard of any such solutions is 2.0% in the younger old age group and 8.7% in the oldest age group (Table 5).

**Table 5:** How many digital health tools have you heard of? (by age groups)

		18–64 years old	65–74 years old	75 + years old
**0**	**n**	**9**	**5**	**16**
	**%**	**0.7%**	**2.0%**	**8.7%**
**1–3**	**n**	**194**	**73**	**66**
	**%**	**15.0%**	**29.9%**	**36.1%**
**4–5**	**n**	**525**	**92**	**69**
	**%**	**40.6%**	**37.7%**	**37.7%**
**6–7**	**n**	**566**	**74**	**32**
	**%**	**43.7%**	**30.3%**	**17.5%**
**Total**	**n**	**1294**	**244**	**183**
	**%**	**100.0%**	**100.0%**	**100.0%**

### Use of digital health solutions

Beside whether they had heard of different types of digital health solutions, respondents were also asked about their use of the above mentioned technologies and devices.

Our results show that around 70% of respondents in both old age groups have used one to three different digital health-related tools (72.8% and 70.3%). The proportion of respondents who have not tried this type of technology is 14.6% and 26.4% respectively.

Most respondents from the 65–74 year age group are familiar with and use e-prescription: 90.2% of respondents are familiar with it and 86.4% of both age groups use it. Around three quarters (77.1%) of 65–74 year olds have heard of and nearly half (45.5%) have used online appointment making. Almost half of the respondents in this age group have heard of and used electronic transmission of medical records and data. A similar proportion had heard about and used apps: 54.3% have heard of them, but only 17.3% have used them. However, in the 75 + age group, significantly fewer have heard of and used online appointment booking (p < 0.003), data/information transfer (p < 0.000) and smart device apps (p < 0.032) (Table 6).

In the 18–64 age group, e-prescription is the most widely known digital health solution (94.1%) which is used by more than three quarters of the respodents, followed by online appointment booking (89.5% and 53.2%) and smart devices with 87.5% awareness and 43.1% usage. The least known tool among the youngest age group is telemedicine (39.2%), used by only 14.8%.

**Table 6:** Awareness and usage of digital health tools – by age groups

	Have you heard about these digital health tools? (by age groups)			If you have heard of them, have you used these digital health tools? (by age groups)		
	18–64 years old	65–74 years old	75 + years old	18–64 years old	65–74 years old	75 + years old
Online appointment booking and referral request	**1161**	**189**	**116**	**618**	**86**	**33**
	**89.7%**	**77.1%**	**63.7%**	**53.2%**	**45.5%**	**28.4%**
Prescription, e-prescription	**1218**	**221**	**156**	**931**	**191**	**127**
	**94.1%**	**90.2%**	**85.7%**	**76.4%**	**86.4%**	**81.4%**
Transmission of data and findings	**933**	**129**	**60**	**553**	**67**	**20**
	**72.1%**	**52.7%**	**33.1%**	**59.3%**	**51.9%**	**32.8%**
Social media related to health information	**647**	**118**	**64**	**214**	**24**	**10**
	**50.0%**	**48.2%**	**35.2%**	**33.1%**	**20.3%**	**15.6%**
Application (e.g.: sleep monitoring, blood sugar diary, symptom diary, etc.)	**948**	**133**	**82**	**287**	**23**	**8**
	**73.3%**	**54.3%**	**45.1%**	**30.3%**	**17.3%**	**9.8%**
Telemedicine	507	97	55	**75**	**23**	**13**
	39.2%	39.6%	30.2%	**14.8%**	**24.0%**	**23.6%**
Smart devices, sensors (e.g.: smart watch, smart scale, pulse oximeter)	**1132**	**167**	**111**	**488**	**29**	**9**
	**87.5%**	**68.2%**	**61.0%**	**43.1%**	**17.4%**	**8.1%**

#### Digital health solution needs

Regarding possible needs for digital health solutions, participants of the survey could choose from the following list: possibility to communicate with their doctor via e-mail; possibility to share images with their doctor through digital channel; possibility to remotely consult with their doctor (Skype or video chat); possibility to share their medical records with their doctor electronically; possibility for their doctor to follow the changes in their health status on their smartphone; possibility to use health sensors at home (systems that measure blood pressure, pulse, body temperature etc.); possibility to browse websites with authentic medical information; possibility to use social media to communicate with their doctor; possibility to book an appointment with their doctor online; possibility for the doctor to recommend an application, sensor, etc.)

Interest in digital technologies is indicated by the fact that only a quarter of 64–75-year-olds (26.5%) and a third of 75 + year olds (31.9%) said they would not like to try digital technologies in the future - i.e. nearly 70% of this age group would like to learn about such tools. This high level of interest is also shown by the fact that more than a fifth of older adults would like to have access to 7–10 of the maximum number of digital devices available.

Among the digital technology needs of both age groups, online appointment booking (51.4% and 39%), home sensors (45.7% and 40.1%), electronic transmission of health records (39.2% and 30.2%), allowing the doctor to track changes in health status digitally (32.7% and 36.8%), having a doctor recommend trustworthy websites (36.7% and 32.4%) and recommending apps (34.3% and 25.3%) are the most important needs.

If these needs are further grouped by their type (online communication, online administration and telemedicine, see details of the grouping in Methodology section), it can be seen that in both age groups, the greatest interest/need is for different telemedicine solutions: 58.8% of those aged 65–74 and 57.1% of those aged 75 + are open to such solutions. Almost 60% of 65–74 year olds demand digital administration, with a lower proportion of 46.7% in the older age group. Online communication solutions with doctors are the least in demand: 53.9% and 45.6% respectively (Table 7).

**Table 7:** Grouped needs for digital health solutions (by age groups)

	18–64 years old	65–74 years old	75 + years old
Digital communication	**1143**	**132**	**83**
	**88.3%**	**53.9%**	**45.6%**
Digital administration	**1176**	**146**	**85**
	**90.9%**	**59.3%**	**46.7%**
Telemedicine	**1110**	**144**	**104**
	**85.8%**	**58.8%**	**57.1%**

#### Perceived advantages and disadvantages of digital health solutions

In terms of positive consequences (Table 8) both age groups have similar views: the biggest advantages are time saving (86.5% and 83.0%), convenience (81.2% and 80.8%) and faster access to care (69.1% and 73.6) (Table 8).

**Table 8:** Perceived positive consequences of digital healthcare solutions for society (by age groups)

	18–64 years old	65–74 years old	75 + years old
Improves the efficiency of care (e.g. you get your care faster)	**1016**	**156**	**116**
	**78.5%**	**63.7%**	**63.7%**
Improves security of care	801	158	123
	61.9%	64.5%	67.6%
It helps patients to cooperate better in the healing process	959	168	131
	74.1%	68.6%	72.0%
Comfortable	**1206**	**199**	**147**
	**93.2%**	**81.2%**	**80.8%**
It reduces the number of personal doctor-patient encounters	**1118**	**187**	**129**
	**86.4%**	**76.3%**	**70.9%**
You can save time with it	**1166**	**212**	**151**
	**90.1%**	**86.5%**	**83.0%**
Patients can access healthcare more quickly	898	170	134
	69.4%	69.1%	73.6%
Doctors involve patients more in the healing process	809	151	127
	62.5%	61.6%	69.8%
You can get better quality care	**599**	**126**	**109**
	**46.3%**	**51.4%**	**59.9%**
It can reduce the chance of malpractice	**467**	**117**	**102**
	**36.1%**	**47.6%**	**56.0%**
Improves doctor-patient communication	900	171	134
	69.6%	69.8%	73.6%

In terms of negative consequences (Table 9), the most common responses were more depersonalisation of care (80.4% and 67.6%), the negative impact of faulty technologies (71.8% and 69.2%) and the increase in the administrative burden on doctors (69.1% and 63.2%). We may however say that the two older age groups have different opinions on several points, with a lower proportion of those aged 75 and over perceiving potential disadvantages, but the difference is not significant (Table 9).

**Table 9:** Perceived negative consequences of digital healthcare solutions for society (by age groups)

	18–64 years old	65–74 years old	75 + years old
The quality of care will be lower	**433**	**102**	**54**
	**33.5%**	**41.6%**	**29.7%**
Makes patients/doctors frustrated (e.g. due to technical difficulties)	**701**	**155**	**97**
	**54.2%**	**63.3%**	**53.3%**
Patient satisfaction decreases	**451**	**127**	**79**
	**34.9%**	**51.8%**	**43.4%**
It can lead to overdiagnosis	672	144	93
	51.9%	58.8%	51.1%
Patients misinterpret the information shared with them about their health	**960**	**172**	**112**
	**74.2%**	**70.2%**	**61.5%**
Faulty technology can endanger patients' recovery	877	176	126
	67.8%	71.8%	69.2%
Personal data is less secure	**732**	**163**	**104**
	**56.6%**	**66.5%**	**57.1%**
Increase in the administrative burden of doctors	**783**	**170**	**115**
	**60.5%**	**69.1%**	**63.2%**
It increases the risk of physician burnout	**545**	**139**	**93**
	**42.1%**	**56.7%**	**51.1%**
Care becomes more impersonal	**990**	**197**	**123**
	**76.5%**	**80.4%**	**67.6%**
Other	**41**	**17**	**3**
	**3.2%**	**6.9%**	**1.6%**

#### What are the sociodemographic variables that may influence the digital health use patterns of the elderly?

When analysing these questions, a methodological limitation was made because of the statistically relevant number of elements, the age group of 65 and over was not split into two categories but analysed as a single unit.

For internet information search, significantly more resources were used by the more highly educated (p < 0.001) than by those living in the capital and county seats (p < 0.018) and by those living with a partner (p < 0.041). The more highly educated had heard of more digital technologies (p < 0.001) and more types of digital solutions were used by the more highly educated (p < 0. 001) and those living in the capital and county seats (p < 0.001). In terms of needs for digital solutions, men (p < 0.014), the more highly educated (p < 0.001) and those living in the capital and county seats (p < 0.020) had significantly more needs for more types of digital technology.

Among those aged 65 and over, there is no significant gender difference in the number of sources of information used or in the amount of digital health technology they have heard about. In terms of digital device use, men were found to have used significantly more devices (p < 0.040). As for digital health needs, men were found to have used significantly more options (p < 0.005).

#### Multivariate analysis

As a final step of the analysis, binary logistic regression was used in order to examine the main background variables for the presence of demand for digital technologies. The model included gender, education, type of settlement, presence of chronic illness and the perceived positive and negative consequences of different digital health solutions. The model is significant overall (Table 10), with a model explained variance (Nagelkerke R^2^) of 26.83%. According to our results, the need for digital solutions increases with the level of educational attainment (high school graduation p < 0.000, OR = 3.093 CI: 1.550–6.175 tertiary education p < 0.001 OR = 8.214 CI 2.382–28.326), and the more benefits one perceives in using digital solutions (p < 0.000, OR: 1.210, CI: 1.131–1.1295). There is also a tendency for the absence of chronic illness to increase the preference (p < 0.095, OR = 1.586, CI: 0.922–2.729 Table 10).

**Table 10:** Logistic regression model for the presence of demand for digital health technologies

Nagelkerke R-square = 0,268								
	B	S.E.	Wald	df	Sig.	Exp(B)	95% C.I.for	
							Lower	Upper
Sex (1 = male; 2 = female)	-0.429	0.274	2.440	1	0.118	0.651	0.380	1.115
Educational level (reference: elementary school)			18.183	3	0.000			
Educational level = vocational school, skilled worker	0.514	0.436	1.393	1	0.238	1.672	0.712	3.929
Educational level = high school	1.129	0.353	10.253	1	0.001	3.093	1.550	6.175
Educational level = higher education	2.106	0.632	11.116	1	0.001	8.214	2.382	28.326
Type of residence (reference = village)			12.207	3	0.007			
Type of residence = other city	0.410	0.404	1.030	1	0.310	1.507	0.683	3.325
Type of residence = county capital, larger city	-0.596	0.358	2.766	1	0.096	0.551	0.273	1.112
Type of residence = capital	0.561	0.299	3.520	1	0.061	1.753	0.975	3.152
Do you have chronic disease (0 = no; 1 = yes)	0.461	0.277	2.780	1	0.095	1.586	0.922	2.729
What do you think are the possible positive consequences of digital healthcare solutions for society?	0.191	0.035	30.502	1	0.000	1.210	1.131	1.295
What do you think are the possible negative consequences of digital healthcare solutions for society?	0.002	0.044	0.003	1	0.955	1.002	0.920	1.092
Constant	-0.660	0.659	1.002	1	0.317	0.517		

Dependent variable: Do you have a need for digital healthcare technology? 0 = no, 1 = yes.

## Discussion

Our study measured the digital health usage habits, attitudes and needs of the 65 and over Hungarian population. Our research, conducted in the 3^rd^ wave of the COVID-19 pandemic (autumn 2021), shows that nearly half of the people aged 65–74 and a third of the people aged 75 + use the internet for health-related purposes (83.5% of people under 65), mostly to access websites. The results of a study on the health related internet use habits of older people in the US [33] also highlights the key role played by education: those with greater than a high school education (75.7%) were more likely to use the Internet than those with high school education or less (40.8%). As noted in the introduction, there is evidence from the EU and China as well that gender disparity is stronger among the elderly than in other age groups. [17]). Elderly women are at an increased risk of missing out on the health advantages digital solutions may offer. Our results also point to the observation that men in this age group are more open to using digital health solutions than women of similar age. This is a shift from the under 65-year group where women prove to be the digital health managers, not men. [33]

In our survey there is a higher proportion of interest and use among those living in the capital and in cities. Our results are confirmed by other data from Hungary. According to a 2018 study reported by the National Media and Infocommunications Authority, almost everyone under the age of forty is an internet user in Hungary, while only over 30% of those over the age of 70 belong to the category of users. Interestingly, among younger people, financial situation does not affect internet use - there is no difference among people of different economic standing. Among older persons, however, educational level and economic situation plays a significant role. More educated older people and those in a better financial situation use the internet much more than other older people. This indicates that internet use among older persons is an economic and cultural issue which can be attended to by providing training opportunities, user friendly programmes and subsidized technology for them [30].

The multivariate analysis showed that those who see more benefits of digital health solutions would demand more of them. This suggests that better information and involvement of older people could be associated with the use of digital tools. Cao et al. examined how the neglect of digital health technologies among older people is evolving. Their research highlighted the importance of communicating benefits and personal connections over the convenience of using digital devices. Education and raising awareness are therefore essential [34]. Although the COVID-19 pandemic has forced almost the general population to use various digital tools, and older adults were no exception, education and support from other participants in the healthcare system is of crucial importance for this population [7]. Our own previous research and our pilot project [35] confirms that well-designed and implemented telemedicine care is feasible for older people experiencing homelessness. The importance of support with the use of technology is highlighted by the fact that a backbone of telemedicine services is the role of institutional assistants, who can effectively support both the doctor’s work and the patient’s orientation in the system.

Another important finding of our study is that the absence of chronic illness tends to be associated with the demand for digital health solutions and, in line with this, those who visit their doctor tend to use fewer internet resources. Trust in doctors is relatively high in both age groups when compared to the under 65 age group, because they are more positive about doctors’ attitudes towards finding information online and less likely to search for information online when seeing a doctor. The higher general level of trust in doctors among older people is in line with the results of other international studies [36–38], indicating that the primary “source” or reference point for this age group is face-to-face contact with the doctor and that digital solutions are of secondary importance. The different levels of eHealth literacy may also play a role: the aforementioned study of older people in the US [33] highlights that fewer of those who preferred to rely on their doctor’s knowledge for medical decision making (38.3%) had high eHealth literacy than those who did not prefer to rely on their doctor’s knowledge (58.6%). However, digital tools can also play an increasingly important role in the care of chronic patients through physician education and the use of relevant tools [39]. A good doctor-patient relationship is a major factor in the effectiveness of digital solutions like telemedicine. This indicates that personal and digital tools can complement each other [40]. Our previous research showed that the homeless population is open to telemedicine use to the same extent as is the domestic reference group. and the degree of openness to the use of telemedicine is primarily a question of trust in the traditional health care system [41].

At the same time, individual and institutional solutions must be implemented to help people in this age group to adapt successfully [42]. Maintaining that with help older adults could navigate the internet, the National Media and Infocommunications Authority launched the ’Netre Fel’ (loosely translated as ’ride the net’) initiative containing easy to use information and guide for older people to use the internet. What is more important, they can ask for help online and can be also put in touch with ‘super-helpers’ volunteers [43].

The strength of our research is that it used a representative sample to explore the digital health habits, needs and attitudes of the Hungarian population 65 years and older. This information is essential for the design of further digital health programmes. However, a limitation of our study is that the relatively small number of items in each category of analysis allows limited conclusions to be drawn. Further research is needed to better understand these trends. It is also a limitation that the question of finding health information on the Internet only examined the availability of sources and did not ask questions about credibility and reliability.

The number of people aged 65 and over globally is 10% of the population, which is expected to increase to 16% by 2050 [44]. The bio-psycho-social approach to health and disease is complemented by a digital component and, in addition to the classic three factors, is of enormous importance in shaping health status [45]. At the same time, getting connected to the digital world, being well informed and competent in the use of tools is a huge challenge for all generations. Our research has shown that the older age group also has measurable needs in digital health, so helping them on this journey is in the interest of the whole health ecosystem. The differences between the two age groups - with younger people being more open and using digital solutions more - and the fact that the under 65s are better adapted in all respects raises the possibility that the specific trends in digital health for older people will virtually disappear in 10 years’ time (when the under 65s now enter this age group). Unless something happens that will make these people stop using the net it may be assumed that as today’s youth are becoming tomorrow’s elderly, internet use will be close to universal in that age group, too [46].

### Electronic supplementary material

Below is the link to the electronic supplementary material.

Supplementary Material 1

Supplementary Material 2

## Data Availability

The datasets used and/or analysed during the current study available from the corresponding author on reasonable request.
