# Supplementary material for: Older adults in the digital health era: insights on the digital health related knowledge, habits and attitudes of the 65 year and older population
Source: BMC Geriatr. 2023 Nov 27;23:779. doi: 10.1186/s12877-023-04437-5 (PMC10683351; doi:10.1186/s12877-023-04437-5)
Supplement: Supplementary file 1 — Supplementary Material 1 [file 12877_2023_4437_MOESM1_ESM.docx]

POPULATION QUESTIONNAIRE

Hello, I'm xxx, contacting you on behalf of the Ipsos Public Opinion Polling Company. In the next few minutes, we ask for your help in one of the first Hungarian surveys about digital health. Your answers will help us see more clearly the opportunities, expectations and limitations related to the use of digital technologies During the survey, we record the answers completely anonymously. Survey data is only analysed statistically in an aggregated manner. Answering is voluntary. Filling out the questionnaire takes about 15 minutes. Would you help me in this?

Sociodemographic data

D1 Which year were you born in?

PROG: Maximum 2003 (IF > 2003 – TERMINATE)

D1 Age group

PROG: THE QUESTION DOESN’T APPEAR, IT IS CODED AUTOMATICALLY FROM THE 3ND QUESTION—UNDER 18—THE INTERVIEW IS OVER

| 1. | 18-29 year old |  |
| --- | --- | --- |
| 2. | 30-39 year old |  |
| 3. | 40-49 year old |  |
| 4. | 50-59 year old |  |
| 5. | 60-69 year old |  |
| 6. | 70 years and older |  |

D2. What is your gender?

| 1. | Male |  |
| --- | --- | --- |
| 2. | Female |  |
| 3. | Other |  |
| 99. | doesn’t know/doesn’t answer | *INTERVIWER: DON’T READ IT* |

D3. What kind of settlement is your permanent residence in?

| 1. | Capital |  |
| --- | --- | --- |
| 2. | County seat |  |
| 3. | Town |  |
| 4. | Village |  |
| 99. | doesn’t know/doesn’t answer | *INTERVIWER: DON’T READ IT* |

D4. Which county do you live in?

Interviewer: don’t read it just mark the answer.

| 1. | Budapest | **PROG: automatically coded if D3=1**  **Ha D3=2,3,4 -> answer option doesn’t appear** |
| --- | --- | --- |
| 2. | Bács-Kiskun |  |
| 3. | Baranya |  |
| 4. | Békés |  |
| 5. | Borsod-Abaúj-Zemplén |  |
| 6. | Csongrád-Csanád |  |
| 7. | Fejér |  |
| 8. | Győr-Moson-Sopron |  |
| 9. | Hajdú-Bihar |  |
| 10. | Heves |  |
| 11. | Jász-Nagykun-Szolnok |  |
| 12. | Komárom-Esztergom |  |
| 13. | Nógrád |  |
| 14, | Pest |  |
| 15. | Somogy |  |
| 16. | Szabolcs-Szatmár-Bereg |  |
| 17. | Tolna |  |
| 18. | Vas |  |
| 19. | Veszprém |  |
| 20. | Zala |  |
| 99. | doesn’t know/doesn’t answer | *INTERVIWER: DON’T READ IT* |

D4a. Region

### PROG: The question doesn’t appear; it is automatically coded from question D4

| 1. | Central Hungary (Budapest, Pest county) | **PROG: D4=1,14** |
| --- | --- | --- |
| 2. | Central Trans-Danubial (Fejér, Komárom-Esztergom, Veszprém) | **PROG: D4= 7, 12, 19** |
| 3. | West Trans - Danubial (Győr-Moson-Sopron, Vas, Zala) | **PROG: D4=8, 18, 20** |
| 4. | South Trans-Danubia (Baranya, Somogy, Tolna) | **PROG: D4=3, 15, 17** |
| 5. | North Hungary(Borsod-Abaúj-Zemplén, Heves, Nógrád) | **PROG: D4=5, 10, 13** |
| 6. | North Alföld (Hajdú-Bihar, Jász-Nagykun-Szolnok, Szabolcs- Szatmár-Bereg) | **PROG: D4=9, 11, 16** |
| 7. | South Alföld -Alföld (Bács-Kiskun, Békés, Csongrád-Csanád) | **PROG: D4=9, 11, 16** |

D5. What is your highest educational attainment?

| 1. | Elementary school or lower |  |
| --- | --- | --- |
| 2. | Vocational school with no school leaving examination |  |
| 3. | Grammar school or vocational school with school leaving exam |  |

| 4. | University/ College (Degree) |  |
| --- | --- | --- |
| 99. | Doesn’t know/ doesn’t want to answer | *INTERVIWER: DON’T READ IT* |

D6. What is your present family status?

| 1. | Single |  |
| --- | --- | --- |
| 2. | Cohabitation or in a lasting relationship |  |
| 3. | Married- lives together |  |
| 4. | Married – lives separately |  |
| 5. | Divorced- |  |
| 6. | Widow/ widower |  |
| 99. | doesn’t know/doesn’t answer | *INTERVIWER: DON’T READ IT* |

D7. How many children under the age of 18 do you have?

## PROG: NUM OE

D8. What is your present labour market status? Please mark the one most characteristic of you!

| 1. | Employed in a leadership position |  |
| --- | --- | --- |
| 2. | Employed in an employee position |  |
| 3. | Self-employed- independent entrepreneur (freelancer or self-employed with no employees) |  |
| 4. | Entrepreneur with employees |  |
| 5. | Pensioner (old age pensioner in their own right, disabled pensioner, widow/widower pensioner) |  |
| 6. | Unemployed |  |
| 7. | Full time student (University, secondary school, training course) |  |
| 8. | Receiving parental support (maternity/ paternity leave) |  |
| 9. | Homemaker, other inactive earner |  |
| 99. | doesn’t know/doesn’t answer | *INTERVIWER: DON’T READ IT* |

# Health Status

Q1. What is your health status like?

*INTERVIWER: don’t read the number of the answer*

| 1. | Very good |  |
| --- | --- | --- |
| 2. | Good |  |
| 3. | Satisfactory | *INTERVIWER: DON’T READ IT ???* |
| 4. | Bad |  |
| 5. | Very bad |  |

Q2. Have you got any long standing illnesses or health conditions?

An illness or health condition is long standing if it lasts at least 6 months or is expected to last at least six months. Examples include hypertension, diabetes, cardiovascular and circulatory illnesses, tumours, locomotor disorders, asthma, allergies

*INTERVIEWER: read all the examples*

| 1. | I have |  |
| --- | --- | --- |
| 2. | I don’t have |  |
| 99. | doesn’t know/doesn’t answer | *! INTERVIWER: DON’T READ IT* |

### PROG: IF Q2 = “I don’t have” or doesn’t answer, go to Q4

Q3: what illness, health impairment do you have?

**PROG: OE**

Q4. Have you ever been tested positively for COVID 19?

| 1. | Yes |  |
| --- | --- | --- |
| 2. | No |  |
| 99. | doesn’t know/doesn’t answer | *INTERVIWER: DON’T READ IT* |

Q5. Are you limited in your daily activates by any illness or impairment (Hearing, sight, movement, mental activities)?

| 1. | Yes, severe activity restrictions |  |
| --- | --- | --- |
| 2. | Yes, I have restrictions, but not serious |  |
| 3. | I have no restrictions |  |
| 99. | doesn’t know/doesn’t answer | *INTERVIWER: DON’T READ IT* |

Q6. In the last 12 months, how often have you used health services either in person or online or telephone?

| 1. | More than once a week |  |
| --- | --- | --- |
| 2. | Weekly |  |
| 3. | More than once a month |  |
| 4. | Monthly |  |
| 5. | A few times a year |  |
| 6. | Yearly |  |
| 7. | Rarer than yearly |  |
| 8. | Never |  |
| 99. | doesn’t know/doesn’t answer | *INTERVIWER: DON’T READ IT* |

# Health related internet use

Q7. Do you use the internet?

| 1. | Yes | **PROG: go to Q8** |
| --- | --- | --- |
| 2. | No | **PROG: jump to Q9** |

Q8. How often do you use the internet to collect health related information?

### PROG: ask if Q7=1 (yes)

| 1. | Daily |  |
| --- | --- | --- |
| 2. | Weekly |  |
| 3. | Monthly |  |
| 4. | Rarer |  |
| 5. | Never | **PROG: Go to Q13** |

Q9. Do your family members, acquaintances help you search for health related information?

### PROG: Ask if Q7=2 (no)

| 1. | No | **PROG: Go to Q10** |
| --- | --- | --- |
| 2. | Yes | **PROG: Jump Q13** |

Q10. Where on the internet do you get health related information or news? More than one answer is acceptable.

## PROG: MA

*INTERVIWER: Read one by one!*

| 1. | Yes |
| --- | --- |
| 2. | No |

| 1. | Websites ( e.g.házipatika, webbeteg) |  |
| --- | --- | --- |
| 2. | Blogs/vlogs (e.g. Funkcionális orvoslás) |  |
| 3. | Online radio, Podcasts |  |
| 4. | Social Media, facebook, instagram etc. (e.g. Novák Hunor, Akut szakasz) |  |
| 5. | Online communities, facebook group, fórums (e.g.  „Cukorbeteg vagyok” facebook csoport |  |
| 6. | YouTube and other video content sharing sights |  |
| 7. | Scientific, professional search engines (e.g. google scholar) |  |

| 8. | Medical databases (f.e. PubMed) |  |
| --- | --- | --- |
| 9. | Medical, Healthcare professional sites and journals |  |
| 10. | Other:… | **PROG: OE** |
| 99. | doesn’t know/doesn’t answer | *INTERVIWER: DON’T READ IT* |

Q11. In your opinion, how do doctors relate to patients who get health information online?

| 1. | 1 They completely oppose it |  |
| --- | --- | --- |
| 2. | 2 |  |
| 3. | 3 |  |
| 4. | 4 |  |
| 5. | 5 – They completely support it |  |
| 99. | doesn’t know/doesn’t answer | *INTERVIWER: DON’T READ IT* |

# Digital health

Q12.Do you use online medical information even in the case if you turn to a doctor?

| 1. | Yes, before seeing a doctor |  |
| --- | --- | --- |
| 2. | Yes, after seeing a doctor |  |
| 3. | Yes, before and after seeing a doctor |  |
| 4. | No |  |
| 99. | doesn’t know/doesn’t answer | *INTERVIWER: DON’T READ IT* |

Q13. From the list below, what is it that you have heard of, what is it that you use and what is it you would like to use for health purposes?

|  |  | Q13.  Have heard of it? | **PROG: If Q13= 1 (yes)**  Q13a. If has heard of it…: | | **PROG: If Q13a= 2 (hasn’t used it yet)**  **)** Q13b. If hasn’t used it yet…: | |
| --- | --- | --- | --- | --- | --- | --- |
|  |  | 1 - Yes/ 2 - No | 1 -  Use it/ has used it before | 2- hasn’t used it yet | 1 – would like to use it | 2 – wouldn’t like to use it |
| 1. | **Online** appointment booking and referral requests |  |  |  |  |  |
| 2. | e-prescription |  |  |  |  |  |
| 3. | transmission of data, results and health records online (EHR) |  |  |  |  |  |

| 4. | Social Media (e.g. Facebook, Instagram) for health related information |  |  |  |  |  |
| --- | --- | --- | --- | --- | --- | --- |
| 5. | Applications eg: sleep monitoring, blood sugar diary, symptom diary, etc. |  |  |  |  |  |
| 6. | Telemedicine: teleconsultations - videos, telephone conversation with the doctor |  |  |  |  |  |
| 7. | Smart devices, sensors (e.g smartwatch, pulseoximeter) |  |  |  |  |  |

Q14. Which of the options below do you use and which would you use if you had the chance?

*Option to ….*

| 1. | uses it/ has used it |
| --- | --- |
| 2. | doesn’t use it but would like to given the chance |
| 3. | doesn1t use it and would not like to use it |

### PROG: randomise

| 1. | Communicating with your doctor via e-mail |
| --- | --- |
| 2. | Sharing images with your doctor through digital channels |
|  | Teleconsultations with your doctor (Skype or video chat) |
| 4. | Sharing health documentations with your doctor electronically |
| 5. | Letting your doctor follow changes in your health status through your smart watch |
| 6. | Using health sensors at home (systems monitoring your blood pressure, pulse, temperature and physical activity complexly and validly |
| 7. | Browsing sites containing valid medical information |
| 8. | Using Social Media in communicating with your doctor |
| 9. | Booking a medical appointment online |
| 10. | Having your doctor recommend apps and sensors for you . |

1. **Potential positive effects of using digital health solutions**

Q15. What do you think may be the potential positive consequences for society from using digital health solutions (e.g. apps on smartphones, smart watches, smart bands, other sensors?

## PROG: Ask everyone

|  |  | Yes | No |
| --- | --- | --- | --- |
| 1. | Improves the convenience of health care (e.g. you can get care faster) |  |  |
| 2. | Improves the safety of care |  |  |
| 3. | Helps involve patients in the process of care |  |  |
| 4. | It is comfortable |  |  |
| 5. | Limits the number of in person doctor-patient |  |  |
| 6. | Saves time |  |  |
| 7. | Patients can get health care quicker |  |  |
| 8. | Patients are more involved in the process of health care |  |  |
| 9. | You can get better quality care |  |  |
| 10. | Limits the possibility of malpractice |  |  |
| 11. | Improves doctor-patient communication |  |  |

### Potential negative effects of using digital health solutions

Q16. What do you think may be the potential negative conswquences for society from using digital health solutions (e.g. apps on smartphones, smart watches, smart bands, other sensors?)

PROG: Ask everyone

## !

|  |  | Igen | Nem |
| --- | --- | --- | --- |
| 1. | Health care becomes worse quality |  |  |
| 2. | It frustrates doctors and patients (e.g. because of technical difficulties) |  |  |
| 3. | Decreases patient satisfaction |  |  |
| 4. | May lead to overdiagnosis ( minor disease is screened may lead to an increase of cases being treated and hence to an increased workload for the healthcare system) |  |  |
| 5. | Patients misinterpret the health data they receive |  |  |
| 6. | Faulty technology may endanger the healing of the patients |  |  |
| 7. | Personal data are not safe |  |  |
| 8. | The administrative burden of doctors increases |  |  |
| 9. | The risk of medical burnout increases |  |  |
| 10. | Health care becomes more impersonal |  |  |
| 11. | Other……: |  |  |

### Personal attitudes with regards to digital health solutions

Q17. What feelings and thoughts do you have with regards to digital health solutions e.g. apps on smartphones, smart watches, smart bands, other sensors?)

## PROG: Ask everyone

| 1. | Very bad |  |
| --- | --- | --- |
| 2. | Bad |  |
| 3. | No feelings are evoked |  |
| 4. | Good |  |
| 5. | Very good |  |
